# Quality Awareness and Its Influence on the Evaluation of App Meta-Information by Physicians: Validation Study

**DOI:** 10.2196/16442

**Published:** 2019-11-18

**Authors:** Urs-Vito Albrecht, Theodor Framke, Ute von Jan

**Affiliations:** 1 Peter L Reichertz Institute for Medical Informatics Hannover Medical School Hannover Germany; 2 Institute for Biometry Hannover Medical School Hannover Germany

**Keywords:** mobile health, evaluation studies, mobile apps, quality principles, usage decisions

## Abstract

**Background:**

Meta-information provided about health apps on app stores is often the only readily available source of quality-related information before installation.

**Objective:**

The purpose of this study was to assess whether physicians deem a predefined set of quality principles as relevant for health apps; whether they are able to identify corresponding information in a given sample of app descriptions; and whether, and how, this facilitates their informed usage decisions.

**Methods:**

All members of the German Society for Internal Medicine were invited by email to participate in an anonymous online survey over a 6-week period. Participants were randomly assigned one app description focusing on cardiology or pulmonology. In the survey, participants were asked three times about whether the assigned description sufficed for a usage decision: they were asked (1) after giving an appraisal of the relevance of nine predefined app quality principles, (2) after determining whether the descriptions covered the quality principles, and (3) after they assessed the availability of detailed quality information by means of 25 additional key questions. Tests for significance of changes in their decisions between assessments 1 and 2, and between assessments 2 and 3, were conducted with the McNemar-Bowker test of symmetry. The effect size represents the discordant proportion ratio sum as a quotient of the test statistics of the Bowker test and the number of observation units. The significance level was set to alpha=.05 with a power of 1-beta=.95.

**Results:**

A total of 441 of 724 participants (60.9%) who started the survey fully completed the questionnaires and were included in the evaluation. The participants predominantly rated the specified nine quality principles as important for their decision (approximately 80%-99% of ratings). However, apart from the practicality criterion, information provided in the app descriptions was lacking for both groups (approximately 51%-92%). Reassessment of the apps led to more critical assessments among both groups. After having familiarized themselves with the nine quality principles, approximately one-third of the participants (group A: 63/220, 28.6%; group B: 62/221, 28.1%) came to more critical usage decisions in a statistically significant manner (McNemar-Bowker test, groups A and B: *P*<.001). After a subsequent reassessment with 25 key questions, critical appraisals further increased, although not in a statistically significant manner (McNemar-Bowker, group A: *P*=.13; group B: *P*=.05).

**Conclusions:**

Sensitizing physicians to the topic of quality principles via questions about attitudes toward established quality principles, and letting them apply these principles to app descriptions, lead to more critical appraisals of the sufficiency of the information they provided. Even working with only nine generic criteria was sufficient to bring about the majority of decision changes. This may lay the foundation for aiding physicians in their app-related decision processes, without unduly taking up their valuable time.

## Introduction

The health app market is highly dynamic and liberally organized, which makes attempts at assessing and adequately regulating it very difficult. There is little reliable information about the actual size of the market, the manufacturers’ composition, or about the products themselves [1]. Various authors lament the deficit of information regarding health apps, apart from marketing-oriented aspects, independent of the platform and area of application [2-5]. There is usually only little information on efficacy, risk profiles, manufacturing processes, and various other key aspects [6-8]. However, in order to make a well-founded decision for usage, as is ethically and legally required for physicians who want to use such health apps in their medical routine, those interested in such apps need sufficient and readily accessible information, ideally provided by the manufacturers; however, there is often a lack of such information. Our own preliminary work confirmed this assumption by means of studying a subset of apps for the cardiological and pulmonological spectrum [9]. Likewise, there are no commonly agreed-upon criteria that those interested in health-related apps, be it for their own use or other purposes such as research, could apply toward assessment of apps in a structured manner [10]; in fact, there is a wide variety of methods that are currently being used. For example, in the context of scientific evaluations, with respect to content, quality is often rated in a descriptive manner or based on surrogate outcome measures [11]. Alternatively, those interested in an app are referred to guidelines and orientation documents, which are more or less extensive catalogues of criteria or third-party certifications or quality seals. These, in turn, also commonly employ variable rating criteria (eg, Ministry of Health New Zealand [12], Canadian Medical Association [13], National Health Service [14], and Fraunhofer-Institut für Offene Kommunikationssysteme [15]; see Albrecht [16] for a more extensive listing of possible approaches). Many such methodologies may be appropriate depending on the context in which they are to be employed. However, some methodologies are possibly either too specific to a certain area of application or confront users with too large a number of criteria and aspects; this makes it difficult for them to determine if the respective approaches are indeed helpful for their use case and whether or not they should use an app.

For new technologies such as mHealth or eHealth in general, there is still a need to survey or at least involve the relevant target groups, in this case physicians, when it comes to creating and evaluating tools offered in the app context or evaluating the apps themselves in line with the requirements of users (eg, Hennemann et al [17], Martínez-Pérez et al [18], and Tarricone et al [19]). This is the case in order to gain acceptance of apps by health care professionals; for this reason, we performed a survey-based evaluation in this paper and fine-tuned our approach to evaluating quality criteria for apps in the health context. In our previous study, we surveyed medical students and demonstrated that there was a considerable discrepancy between the high-quality requirements for health apps, as communicated by the students, and the information provided about these prerequisites in the app descriptions. The study also showed that after examining nine quality principles and 20 features and criteria, students became more critical of the information available in app descriptions. In 3 out of 4 (75%) cases examined, the students changed their opinions regarding the suitability of the app descriptions from *sufficient* or *do not know* to *insufficient*, for the purpose of making their decisions about usage.

In this study, we aimed to validate this approach among physicians. Our main objective was to investigate whether being made aware of, and working with, quality principles, as introduced previously [20], influences physicians' assessments of whether the information in app descriptions was sufficient for them to make a decision about app usage. Furthermore, the basic assessment of the relevance of quality principles for usage decisions by the physicians was also investigated. In addition, we analyzed how physicians assess the sufficiency of the information provided in the app descriptions regarding their compliance with the quality principles. Finally, the frequency of specific aspects, identified by key questions within the assigned app descriptions, was collected in a descriptive manner.

## Methods

### Overview

This study was conducted as an anonymous, investigator-blinded, randomized, and standardized Web-based survey with health care professionals who are members of the German Association for Internal Medicine (ie, Deutsche Gesellschaft für Innere Medizin [DGIM] eV). The survey was conducted at Hannover Medical School using a local installation of the SoSci Survey [21] tool, version 3.2.000 (SoSci Survey GmbH), and was open for a period of 6 weeks, between June 17 and July 29, 2019. On July 8, 2019, an email reminder was sent to all DGIM members who were initially invited to participate. Altogether, approximately 21,000 DGIM members were invited to participate using the DGIM email list. The study was approved by the Institutional Review Board of Hannover Medical School (study number: 8256_BO_K_2019).

### Structure of the Survey

**Figure 1:**
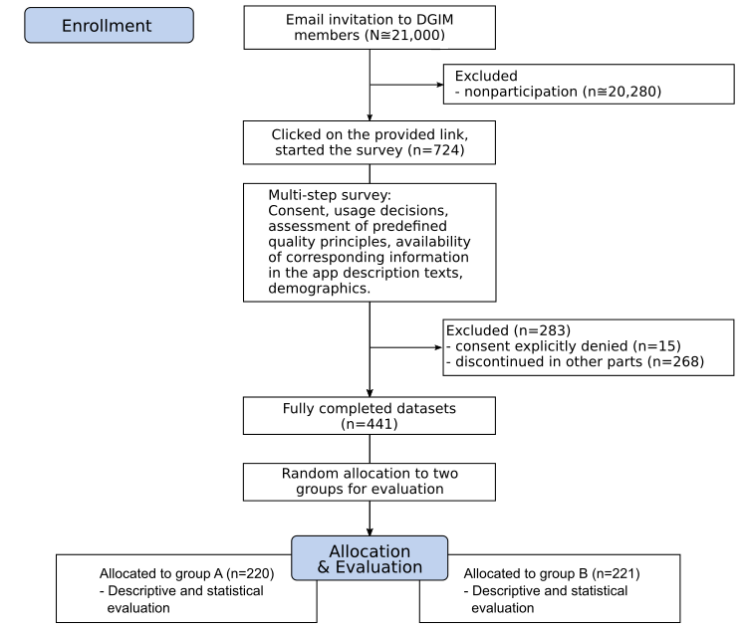
Study flowchart. DGIM: Deutsche Gesellschaft für Innere Medizin.

After a short introduction to the study and obtaining informed consent, participants were asked to evaluate one randomly assigned app description; these descriptions were taken from a selection of 126 cardiology- and pulmonology-related apps acquired from Apple’s app store in 2018, which were used as basis for our previous study [9]. Initially, they were asked to assess whether, in their opinion, the information presented in the store description was sufficient for deciding for or against using the selected app (see Figure 1, assessment 1 [A1]). Subsequently, the study participants were questioned about their perception of the relevance of nine given quality principles for making a decision about usage (see Figure 1, questionnaire 1 [Q1]). This was followed by an assessment as to whether the information contained in the descriptions could be used to conclude that these quality principles were met (see Figure 1). The respondents were then asked to reassess whether the descriptive texts were sufficient for making a decision about usage (see Figure 1). Afterward, the study participants had to determine whether the relevant information was present using closed-ended questions (see Figure 1). Thereafter, analogous to the initial assessment, the participants were prompted to reassess whether the descriptive texts were sufficient for making a decision about usage (see Figure 1). The methodology described is based on a preliminary study with medical students, which has been successfully implemented [9].

### Evaluation Strategy

#### Overview

For validation purposes, the respondents were randomly assigned into two sample groups: A and B. For this purpose, the random number generator from R, version 3.5.2 (The R Foundation), was used to generate and assign random floating-point numbers between 1 and 441 to the fully completed datasets. The dataset rows were then sorted using these numbers. The first dataset members (N=220) were placed in group A and the rest (N=221) were placed in group B. Primary and secondary analyses were performed equally for samples A and B.

#### Primary Analysis

The primary aim was to determine whether there was a change in participants’ answers regarding the assessments done at different stages of the survey (ie, assessments 1, 2, and 3 [A1, A2, and A3]).

To this end, two comparisons were made. The first comparison between the first and second assessments (ie, A1 and A2) quantified the change in assessment after getting to know the quality principles. The second comparison of the second and third assessments (ie, A2 and A3) quantified the change in assessment after participants had checked the app descriptions regarding specific (ie, operationalized) quality principles. For testing, the McNemar-Bowker test [22,23] was used, which considers the first comparison and then the second comparison in the sense of a test hierarchy. The study was regarded as successful if at least the first comparison (ie, A1 vs A2) showed a significant difference according to the research hypothesis. For each comparison, a two-sided hypothesis—H_0_: p_ij_=p_ji_ for all i≠j versus H_1_: p_ij_≠p_ji_ for at least one i≠j—was used (see Table 1). The effect size represents the discordant proportion ratio sum (DPRS) as a quotient of the test statistics of the Bowker test and the number of observation units. The significance level was set to alpha=.05.

**Table 1:** Probabilities of the before-and-after paired decisions (eg, A1^a^ vs A2^b^).

Rating before (eg, A1)	Rating after (eg, A2)			
	Insufficient	Do not know	Sufficient	Total
Insufficient	p_11_^c^	p_12_	p_13_	p_1_
Do not know	p_21_	p_22_	p_23_	p_2_
Sufficient	p_31_	p_32_	p_33_	p_3_
Total	p_1_	p_2_	p_3_	1

^a^A1: assessment 1.

^b^A2: assessment 2.

^c^Proportions p_ij_ are derived by dividing the counts by the total number of participants.

#### Secondary Analysis

In order to analyze the influence of the individual quality aspects on the intention to make a usage decision, a logistic regression with a univariate approach was carried out with a modeled probability of *negative changes* (ie, changes to *do not know* or *insufficient*.)

Furthermore, in a descriptive form, the health care professionals' assessments of the relevance of the quality principles for their usage decision were recorded. The same procedure was used to evaluate the sufficiency of the information provided in the app descriptions in order to assess compliance with the quality principles. In addition, the frequencies of the aspects identified in the descriptions, based on the key questions, were noted.

### Sample Size Calculation

Sample size planning was done as described for the Power Analysis and Sample Size (PASS) software (NCSS) [24] and by Chow et al [25] and was carried out with R, version 3.5.2 (The R Foundation). For a preliminary study with a similar setup that was conducted previously, a DPRS of 0.13 had been calculated. As a result of conservative planning, a DPRS of 0.1 was assumed for this project. This resulted in a sample size of 175 for alpha=.05 and 1-beta=.95 per end point.

### Tools

#### Questionnaires Used at Different Stages of the Survey

Several tools were used for the survey, which was implemented online by means of the server-based SoSci Survey tool, version 3.2.000 (SoSci Survey GmbH) [21]. Q1 and questionnaire 2 (Q2) matched those that were used in our previous study. The questions used for questionnaire 3 (Q3), however, were based on the technical report presented in Albrecht [20]. For the initial assessment (ie, Q1) of the quality principles, which included nine items, a 5-point Likert scale with an additional field for *do not know* was used and the answers were mandatory (see Table 2).

Using nine questions with possible answers of *yes*, *no*, and *do not know*—answers were mandatory; see Table 3 for the corresponding questions—participants were asked via Q2 whether there was sufficient information in the app descriptions to ascertain whether the apps met these principles.

**Table 2:** The nine quality principles and their explanations used for the initial assessment (ie, Q1^a^), following the definitions in Albrecht et al [9].

Quality principle ID	Explanation
Q101	Practicality: The software can be used for the intended purpose and must be as versatile as possible in order to open up the largest possible application areas and contexts.
Q102	Risk adequacy: The software provides the means to be used in a risk-appropriate manner without exposing the user or his or her environment to a disproportionate health, social, or economic risk.
Q103	Ethical soundness: Development, provision, operation, and use of the software are ethically innocuous in order to prevent discrimination and stigmatization and to facilitate fair access.
Q104	Legal conformity: Legal conformity (eg, medical device law, professional law, data protection law, and law on the advertising of therapeutic products) of the development, provision, operation, and use of the software is guaranteed for the protection of all parties involved (eg, providers, store operators, and users).
Q105	Content validity: The health-related content of the software that is presented and used is valid and trustworthy (ie, scientifically sound, up-to-date, and without conflict of interest).
Q106	Technical adequacy: Development, operation, maintenance, and use of the software correspond to the state of the art in order to enable sustainability in terms of maintainability as well as platform-independent and cross-platform use (eg, in terms of portability of the app or interoperability or compatibility with other products).
Q107	Usability: The software allows the target group to make appropriate use of it (eg, through product ergonomics, accessibility, and aesthetics), which contributes to user satisfaction.
Q108	Resource efficiency: During development of the software, elements for resource-efficient operation (eg, energy consumption) and use (eg, computing time) are taken into account.
Q109	Transparency: Complete transparency regarding the quality principles serves as a basis for evaluations of the software as well as for individual and collective usage decisions.

^a^Q1: questionnaire 1.

**Table 3:** Questions, via Q2^a^, used for determining whether the presented app descriptions contained information related to the nine quality principles, following those employed in Albrecht et al [9].

Question ID	Question
Q201	Practicality: Can you use the app description to make an assessment as to whether the app is useful?
Q202	Risk adequacy: Can you use the app description to make an assessment as to whether the app is risk adequate?
Q203	Ethical soundness: Can you use the app description to make an assessment as to whether the app is ethically safe?
Q204	Legal conformity: Can you use the app description to make an assessment as to whether the app is legally compliant?
Q205	Content validity: Can you use the app description to make an assessment as to whether the content of the app is valid?
Q206	Technical adequacy: Can you use the app description to make an assessment as to whether the app is technically appropriate?
Q207	Usability: Can you use the app description to make an assessment as to whether the app is usable?
Q208	Resource efficiency: Can you use the app description to make an assessment as to whether the app is resource efficient?
Q209	Transparency: Can you use the app description to make an assessment as to whether the app description is transparent on the above points?

^a^Q2: questionnaire 2.

Following this, a more detailed retrieval of information, via Q3 and using a set of 25 questions, was employed. This tool operationalizes the nine quality principles with 25 features and requirements based on the catalogue of criteria for self-declaration of health apps presented in Albrecht [20]. Answer options in this case were *yes*, *no*, *do not know*, and *not answered*. Although in this case, answers were not defined as mandatory; if any answers were missing in this part of the survey, the participants were alerted once and asked whether they really did not want to rate the corresponding questions before being allowed to continue. There was also a free-text field for optional comments (see Table 4). The interspersed usage decision question (see steps A1-A3) had to be answered using the options *yes*, *no*, and *do not know*; answering this question was mandatory. Lastly, participant demographics (D0) were obtained using 13 items; again, answers were not considered mandatory.

**Table 4:** Operationalization of the nine quality principles using 25 detailed questions, via Q3^a^, according to Albrecht [20].

Question ID	Question	Contribution
Q301	Has the purpose of the app been specified?	Practicality
Q302	Is there a description of functions the app offers in order to fulfill its purpose?	Practicality
Q303	Is there a description of the app given that states which methods it employs to fulfill its purpose (eg, procedures, processes, and algorithms with which the offered functions are implemented), and are there statements regarding their suitability for this purpose?	Practicality
Q304	Is appropriate evidence cited to support the statements on fulfillment of the purpose of the app (eg, references to studies, guidelines, testing, and quality labels)?	Practicality
Q305	Are the suitability and unsuitability for certain app scenarios or user groups specified (eg, in terms of inclusion and exclusion criteria)?	Practicality
Q306	Have potential or actual risks the app poses to users or their environment been stated, with respect to health, economic, or social aspects?	Risk adequacy
Q307	Have precautions been taken to avoid health, economic, and/or social risks when using the described app?	Risk adequacy
Q308	Is there a description about the extent to which the app follows ethical principles, such as patient autonomy, equity of access, and/or professional ethics and research ethics?	Ethical soundness
Q309	Are conflicts of interest (eg, authors with affiliations to specific companies) discussed in the app description?	Ethical soundness
Q310	Is there a mention of whether the app is being provided in a research context; if so, is there a statement about whether it follows good scientific practice?	Ethical soundness
Q311	Is there a statement about whether the relevant general legal requirements, such as data protection law, telemedia law, and commercial law, have been taken into account by the manufacturer and provider of the app?	Legal conformity
Q312	Is there a statement about which requirements and regulations have been taken into account with regard to using the app in a health context, such as medical device law or medical professional law?	Legal conformity
Q313	Is there a statement about how the quality of the content has been ensured (eg, involvement of experts in the field) or which validated sources have been used (eg, consideration of current scientific findings and guidelines)?	Content validity
Q314	Is there a description of how the app is regularly adapted to new content requirements?	Content validity
Q315	Is it described to what extent the app corresponds to the current state of the art?	Technical adequacy
Q316	Is there information about how the app is regularly adapted to technical requirements?	Technical adequacy
Q317	Is there information about to what extent it is possible to switch to another operating system or device without data loss?	Technical adequacy
Q318	Is there a mention of whether the app is scalable (ie, adaptable to increasing requirements) or can be integrated into other products?	Technical adequacy
Q319	Has information been provided about proof for the app's usability (eg, usability tests)?	Usability
Q320	Is it described to what extent the function of the app has been specifically adapted to the target group, whether it is barrier-free, or whether it can be used with individual adaptations?	Usability
Q321	Is there information about the extent to which user feedback was considered for the app (eg, during the development process)?	Usability
Q322	Is there a statement about how the app ensures efficient use of the available technical resources (eg, required memory, computing power, internal or external sensors, and power consumption)?	Resource efficiency
Q323	Is the information about the app sufficient (ie, adequately specified in scope and depth of information)?	Transparency
Q324	Is valid (ie, complete and reliable) information about the app provided?	Transparency
Q325	Is the information about the app described in a manner that is adequate for the target group?	Transparency

^a^Q3: questionnaire 3.

#### Pretest of the Questionnaires

The standardized questionnaires described above were pretested in three iterations. After the creation of the first version, the questionnaire was tested by three medical students on May 22, 2019. This was accompanied by the *thinking aloud* approach, with utterances being used as feedback and a basis for adaptations to the survey (ie, iteration 1). The three students were also asked to paraphrase the questions contained in the survey, to ascertain whether they understood the questions correctly. This made it possible to identify and adapt unclear questions and answer options. In addition, usability issues were addressed. The provided feedback was used for revising the questions. The follow-up version of the questionnaire was then tested by 10 public health students and two lecturers between May 23 and June 6, 2019. This was meant as a pretest under real-life conditions; we used a live version of the questionnaires in our SoSci Survey setup, albeit with an additional comment function (ie, iteration 2). Afterward, solutions for issues discovered in this second pretest, as well as any constructive remarks obtained from the participants, were incorporated into the survey. This was once again presented and discussed with the testers in a follow-up meeting on June 6, 2019 (ie, iteration 3). After any remaining minor issues that were mentioned in this final meeting had been resolved, the questionnaire was finalized.

#### Software Used in the Evaluation Process

The following software programs were used at various stages of the evaluation process to generate graphics as well as for data description and statistics: IBM SPSS Statistics for Mac OS, version 26.0 (IBM Corp), and R, version 3.5.2 (The R Foundation), along with the R packages dplyr [26], ggplot2 [27], RColorBrewer [28], arsenal [29], qwraps2 [30], Hmisc [31], DescTools [32], and rcompanion [33].

## Results

### Response Rates

Out of a total of 1357 clicks on the provided survey link, 724 participants started the questionnaire and 441 of these participants (60.9%) fully completed the questionnaire (see Figure 1 and Table 5 for further details). An additional 15 participants did not consent to participation on the first page of the survey and were, thus, not shown the actual survey questions; instead, they were asked if they were willing to provide information about their reasons for not consenting. Of those who gave a reason for nonparticipation, contributing factors were lack of knowledge about apps (n=6), information about the survey being perceived as insufficient (n=3), lack of interest in the subject (n=2), and lack of time (n=2). Additionally, other reasons given in free-text form were related to being too old and/or being retired (n=3). One additional participant gave a statement to the effect of being worried that her answers would not be sufficiently anonymized.

**Table 5:** Participant dropout at different stages of the survey, from 724 participants who originally started the survey.

Survey page number	Description	Full completion of survey^a^, n	Dropouts, n
9	Questions about reasons for not consenting; these were for participants who had not consented to participation on page 1	15	0
8	Demographics	441	1
7	A3^b^: Final usage decision	N/A^c^	1
6	Q3^d^: In-depth evaluation based on 25 key questions	N/A	16
5	A2^e^: Intermediate usage decision	N/A	45
4	Q2^f^: Assessment of whether the available information suffices for assessing the app based on the nine quality principles	N/A	11
3	Q1^g^: Participants’ assessments of nine predefined quality principles	N/A	44
2	A1^h^: Initial usage decision	N/A	59
1	Introduction and consent	N/A	91
N/A	Sum of participants	456	268

^a^Participants were considered to have completed the survey either by completing the final demographics questions on page 8 or, for those who had not consented to participation, by providing reasons for not giving their consent on page 9.

^b^A3: assessment 3.

^c^Not applicable.

^d^Q3: questionnaire 3.

^e^A2: assessment 2.

^f^Q2: questionnaire 2.

^g^Q1: questionnaire 1.

^h^A1: assessment 1.

### Demographics of the Test and Validation Samples

Baseline demographics for the participants, stratified by the randomly assigned groups A (test sample) and B (validation sample), are shown in Table 6. Table 7 provides additional data about the participants’ interests in digital topics and app usage patterns in private- and work-related areas. For all variables in these two tables, there were only statistically insignificant differences between the two groups. *P* values were calculated using the χ² test function provided by R [34].

**Table 6:** Baseline demographics of all participants who completed the questionnaire; demographics are stratified by gender for the randomly assigned groups A and B.

Baseline demographic (D0)		Group A (N=220), n (%)	Group B (N=221), n (%)	χ²	df	*P* value
**D002: Gender**				**3.2**	**2**	**.20**
	Male	113 (51.4)	130 (58.8)			
	Female	104 (47.3)	90 (40.7)			
	Diverse	3 (1.4)	1 (0.5)			
**D001: Age in years**				**3.9**	**4**	**.42**
	21-30	11 (5.0)	8 (3.6)			
	31-40	60 (27.3)	79 (35.7)			
	41-50	55 (25.0)	48 (21.7)			
	51-60	59 (26.8)	53 (24.0)			
	>60	35 (15.9)	33 (14.9)			
**D003: Years of work**				**7.6**	**6**	**.27**
	<1	2 (0.9)	0 (0)			
	1-5	18 (8.2)	18 (8.1)			
	6-10	33 (15.0)	49 (22.2)			
	11-20	66 (30.0)	57 (25.8)			
	21-30	53 (24.1)	42 (19.0)			
	>30	34 (15.5)	40 (18.1)			
	Retired	14 (6.4)	15 (6.8)			
**D004: Specialty**				**28.0**	**26**	**.37**
	Internal medicine	76 (34.5)	77 (34.8)			
	Internal medicine and general medicine, without further specification	7 (3.2)	17 (7.7)			
	Internal medicine and angiology	5 (2.3)	1 (0.5)			
	Internal medicine and endocrinology and diabetology	6 (2.7)	4 (1.8)			
	Internal medicine and gastroenterology	22 (10.0)	25 (11.3)			
	Internal medicine and hematology and oncology	14 (6.4)	11 (5.0)			
	Internal medicine and cardiology	17 (7.7)	27 (12.2)			
	Internal medicine and nephrology	13 (5.9)	16 (7.2)			
	Internal medicine and pulmonology	12 (5.5)	9 (4.1)			
	Internal medicine and rheumatology	5 (2.3)	3 (1.4)			
	General practitioner	28 (12.7)	19 (8.6)			
	Other specialties	15 (6.8)	12 (5.4)			
**D005: Function at work**				**3.0**	**5**	**.71**
	Chief physician	18 (8.2)	12 (5.4)			
	Senior physician	50 (22.7)	56 (25.3)			
	Junior physician	49 (22.3)	55 (24.9)			
	Student	1 (0.5)	0 (0)			
	Other	99 (45.0)	95 (43.0)			
	No data	71 (32.3)	71 (32.1)			
**D006: Sector of work**				**0.7**	**3**	**.87**
	Outpatient sector	76 (34.5)	76 (34.4)			
	Clinic	120 (54.5)	116 (52.5)			
	Other	23 (10.5)	27 (12.2)			
	No data	24 (10.9)	29 (13.1)			
**D007: Type of employment**				**2.3**	**3**	**.51**
	Salaried	157 (71.4)	148 (67.0)			
	Self-employed	52 (23.6)	56 (25.3)			
	Other	11 (5.0)	16 (7.2)			
	No data	0 (0)	1 (0.5)			
**D008: Highest academic degree**				**6.2**	**6**	**.41**
	Habilitation	15 (6.8)	23 (10.4)			
	Doctoral degree	126 (57.3)	133 (60.2)			
	State exam	71 (32.3)	55 (24.9)			
	Master’s degree, diploma, or similar	4 (1.8)	5 (2.3)			
	Bachelor’s degree or similar	0 (0)	1 (0.5)			
	Other	3 (1.4)	4 (1.8)			
	No data	1 (0.5)	0 (0)			
D009: Geographic location^a^				22.0	31	.89

^a^The data for the individual subcategories (on a federal state level for Germany, Austria, and Switzerland, as well as per country for other members of the European Union) is intentionally not shown here for brevity reasons. It is, however, available upon request from the authors.

**Table 7:** Interest in digital topics and app usage patterns, stratified by randomly assigned groups A and B.

Interest demographic (D0)		Group A (N=220), n (%)	Group B (N=221), n (%)	χ²	df	*P* value
**D010: Interest in digital topics**				**0.2**	**4**	**>.99**
	Highly interested	68 (30.9)	68 (30.8)			
	Interested	95 (43.2)	98 (44.3)			
	Partly interested	41 (18.6)	38 (17.2)			
	Less interested	12 (5.5)	13 (5.9)			
	Not interested	4 (1.8)	4 (1.8)			
**D011: Private use of apps**				**1.8**	**1**	**.18**
	Yes	201 (91.4)	210 (95.0)			
	No	19 (8.6)	11 (5.0)			
**D012: Use of apps at work**				**0**	**1**	**>.99**
	Yes	153 (69.5)	154 (69.7)			
	No	67 (30.5)	67 (30.3)			
**D013: Have been asked about apps by patients**				**0.001**	**1**	**.97**
	Yes	68 (30.9)	67 (30.3)			
	No	152 (69.1)	154 (69.7)			
**A303^a^: App or its description was known**				**3.4**	**2**	**.18**
	Yes	12 (5.5)	5 (2.3)			
	No	206 (93.6)	215 (97.3)			
	Do not know	2 (0.9)	1 (0.5)			

^a^This question was shown on page 7 of assessment 3 (A3) and was not part of the demographics part of the survey on page 8; therefore, it was encoded differently.

### Baseline Assessment of the Nine Predefined Quality Principles

**Figure 2:**
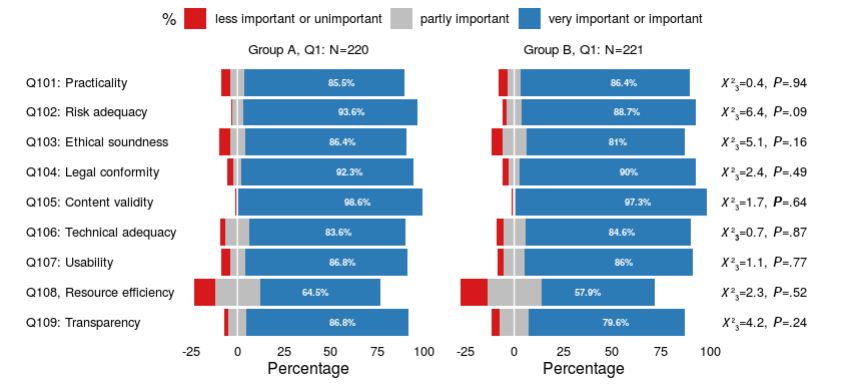
Participants’ assessments, stratified by group, of the perceived importance of the nine quality principles, including corresponding results for the chi-square tests that only show statistically insignificant differences between groups. Q1: questionnaire 1.

In their initial assessment of the presented quality principles—see Table 2 for the definition of the nine quality principles—the majority of participants rated the criteria as *very important* or *important* (80%-99% of the participants). However, there was one notable exception: for resource efficiency, only about two-thirds of the participants perceived this criterion as *important* or *very important*, while around 1 in 4 participants rated it as partially important. As for the demographic variables listed in Table 7, there were again no statistically significant differences between the groups regarding their assessment of the nine quality principles (see Figure 2; Multimedia Appendix 1 provides further information about the answers given by the participants).

### Do the App Descriptions Provide Sufficient Information?

#### Availability of Information That Covers the Nine Quality Principles

**Figure 3:**
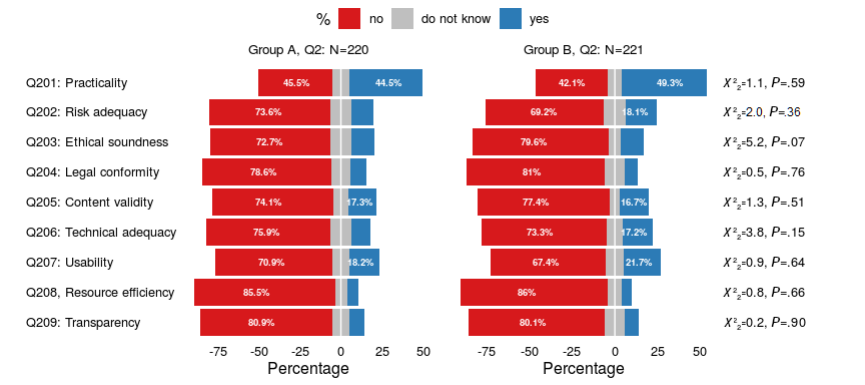
Graphical representation of the participants’ assessments of whether the provided app descriptions contain sufficient information with respect to the nine quality principles, stratified by the randomly assigned groups: group A (test) and group B (validation). Results for the corresponding chi-square tests are also shown. Q2: questionnaire 2.

With the exception of the practicality principle, based on the questions presented in Table 3, the participants were only rarely able to find sufficient information covering the nine quality principles (see Figure 3). For practicality, there was almost an equilibrium between positive versus negative ratings in both groups; for all other aspects, negative ratings in both groups prevailed, with rates ranging from 70% to more than 80%. As shown by the results of the chi-square test shown in Figure 3, there were only negligible, statistically insignificant differences between test group A and validation group B (see Multimedia Appendix 2 for a more detailed listing of the results for Q2).

#### Assessment of the Quality Principles Using 25 Key Questions

**Figure 4:**
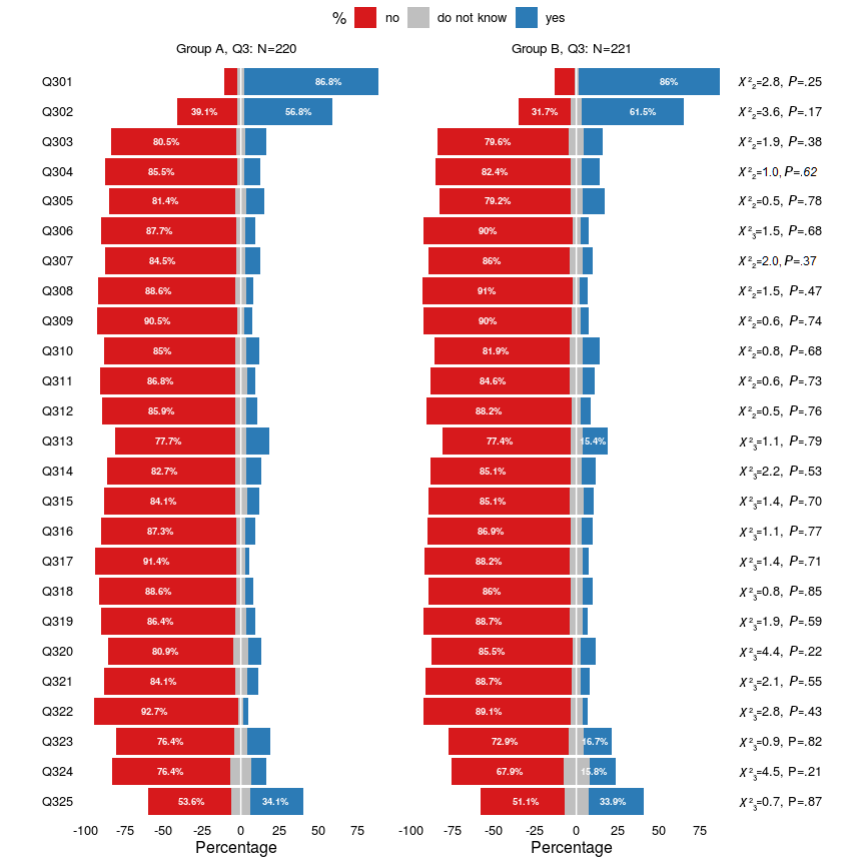
Graphical representation of the participants’ assessments of whether the provided app descriptions contain sufficient information with respect to the nine quality principles, this time assessed using 25 key questions (Q301-Q325) and stratified by the two randomly assigned groups: group A and group B. Results of the chi-square tests are also provided. Q3: questionnaire 3.

Figure 4 is a graphical representation of the participants’ assessments of whether the provided app descriptions contain sufficient information with respect to the nine quality principles. Assessments were made using 25 key questions (Q301-Q325) and were stratified by the two randomly assigned groups: group A and group B. The figure also shows results of the chi-square tests. Detailed results for Q3 are listed in Multimedia Appendix 3.

### Does the Confrontation With the Quality Principles Influence Participants’ App Usage Decisions?

#### Differences Between the Initial Usage Decisions (Assessment 1) and the Intermediate Assessments (Assessment 2)

As shown in Table 8, after having worked with the nine quality principles in steps involving Q1 and Q2 (see Figure 1, as well as Tables 2 and 3 for reference), based on the descriptions, the participants perceived the apps they were confronted with in a more critical manner. For group A, assessments from 63 out of 220 participants (28.6%) changed in a negative direction, both in A1 (ie, from *do not know* to *insufficient*) and in A2 (ie, from *sufficient* to either *do not know* or *insufficient*). For group B, the amount of assessment change in a negative direction was similar (62/221, 28.1%). For both groups, based on the results of the McNemar-Bowker test (see Table 9), these changes were statistically significant (*P*<.001 for both groups).

**Table 8:** Presentation of the contingency table (A1^a^ vs A2^b^) before and after the clarification of quality principles and the targeted search for these quality principles.

A1 (before)	A2 (after): Group A (N=220), n (%)				A2 (after): Group B (N=221), n (%)			
	Insufficient	Do not know	Sufficient	Total	Insufficient	Do not know	Sufficient	Total
Insufficient	97 (44.1)	2 (0.9)	5 (2.3)	104 (47.3)	91 (41.2)	2 (0.9)	2 (0.9)	95 (43.0)
Do not know	10 (4.5)	4 (1.8)	1 (0.5)	15 (6.8)	10 (4.5)	3 (1.4)	1 (0.5)	14 (6.3)
Sufficient	48 (21.8)	5 (2.3)	48 (21.8)	101 (45.9)	42 (19.0)	10 (4.5)	60 (27.1)	112 (50.7)
Total	155 (70.5)	11 (5.0)	54 (24.5)	220 (100)	143 (64.7)	15 (6.8)	63 (28.5)	221 (100)

^a^A1: assessment 1.

^b^A2: assessment 2.

**Table 9:** McNemar chi-square, Cohen g, and odds ratio (OR): assessment 1 vs assessment 2.

Group	Statistic					
	McNemar χ²_3_			Cohen g^b^(df=3)		
	DPRS^a^	Test statistic	*P* value	OR^b^	*P* value	g
Group A (N=220)	0.195	42.89	<.001	7.9	.89	0.39
Group B (N=221)	0.222	49.06	<.001	12.4	.93	0.42

^a^DPRS: discordant proportion ratio sum.

^b^Calculated with the R package rcompanion.

#### Differences Between the Intermediate Usage Decisions (Assessment 2) and the Final Assessments (Assessment 3)

The subsequent application of the quality principles based on the more elaborate 25 key questions (see Table 4 for reference) had considerably less influence on the final usage decision, as depicted by the changes from decisions given in A2 to those given in A3. Here, an increase in critical ratings was only observed for 25 out of 220 participants (11.4%) from group A, and for 20 out of 221 participants (9.0%) from group B. However, these changes were statistically insignificant (see Tables 10 and 11).

**Table 10:** Presentation of the contingency table (A2^a^ vs A3^b^) before and after the clarification of quality principles and the targeted search for these quality principles.

A2 (before)	A3 (after): Group A (N=220), n (%)				A3 (after): Group B (N=221), n (%)			
	Insufficient	Do not know	Sufficient	Total	Insufficient	Do not know	Sufficient	Total
Insufficient	145 (65.9)	3 (1.4)	7 (3.2)	155 (70.5)	136 (61.5)	1 (0.5)	6 (2.7)	143 (64.7)
Do not know	9 (4.1)	1 (0.5)	1 (0.5)	11 (5.0)	2 (0.9)	6 (2.7)	7 (3.2)	15 (6.8)
Sufficient	14 (6.4)	2 (0.9)	38 (17.3)	54 (24.5)	16 (7.2)	2 (0.9)	45 (20.4)	63 (28.5)
Total	168 (76.4)	6 (2.7)	46 (20.9)	220 (100)	154 (69.7)	9 (4.1)	58 (26.2)	221 (100)

^a^A2: assessment 2.

^b^A3: assessment 3.

**Table 11:** McNemar chi-square, Cohen g, and odds ratio (OR): assessment 2 vs assessment 3.

Group	Statistic					
	McNemar χ²_3_			Cohen g^b^(df=3)		
	DPRS^a^	Test statistic	*P* value	OR^b^	*P* value	g
Group A (N=220)	0.026	5.67	.13	2.3	.69	0.19
Group B (N=221)	0.035	7.66	.05	2.8	.73	0.23

^a^DPRS: discordant proportion ratio sum.

^b^Calculated with the R package rcompanion.

#### Correlations and Regression

We were interested in determining whether there were certain factors exerting an influence on app usage decisions (eg, participants’ demographics or the availability of information about the nine quality principles within the app descriptions). We defined A1→A2 as a binary variable, where 1 represented participants with a *negative* change (ie, *sufficient* to *do not know*, *sufficient* to *insufficient*, and *do not know* to *insufficient*) and 0 represented *no* change or *other* changes. As described in the previous two subsections, significant changes for the usage decisions were only noted between the initial (A1) and intermediate assessments (A2). Therefore, we applied the chi-square test for demographics versus change in usage decision (ie, demographics vs A1→A2; see Table 12) and for availability of information corresponding to the nine quality principles (ie, quality principle vs A1→A2; see Table 13). Regarding demographics, with the exception of education in group B (*P*=.04), there was no statistically significant influence (see Table 12).

For availability of information about the nine quality principles versus the change in usage decision, results differed between groups. For group A, there was a correlation between change in usage decision and the quality principles *practicality* (*P*=.01), *risk adequacy* (*P*<.001), and *ethical soundness* (*P*=.01); however, there was only an insignificant influence of these principles on change in usage decision for group B. In contrast, *content validity* (*P*=.03), *resource efficiency* (*P*=.02), and *transparency* (*P*=.01) correlated in a statistically significant manner with participants’ changed usage decisions in group B; however, this was not the case for group A (see Table 13). Detailed results of the regression analysis can be found in Multimedia Appendix 4.

**Table 12:** Demographic factors versus changes in assessment: initial to intermediate assessment (ie, changes from A1^a^ to A2^b^ toward a more critical assessment).

Demographic (D0) vs A1→A2	Group A			Group B			
	χ²	df^c^	*P* value		χ²	df^c^	*P* value
D001: Age	0.5	4	.97		6.7	4	.15
D002: Gender	5.2	2	.08		0.9	2	.63
D003: Years of work	4.3	6	.64		7.2	5	.21
D004: Specialty	26.0	21	.22		10.0	20	.96
D005: Function	2.7	5	.74		6.3	4	.18
D006: Sector	0.5	3	.92		3.6	3	.31
D007: Type of employment	1.2	2	.54		2.8	3	.43
D008: Education	3.1	5	.68		12.0	5	.04
D009: Location	31.0	26	.22		22.0	23	.53
D010: Interest in digital topics	5.8	4	.21		5.2	4	.27
D011: Private app use	1.2	1	.27		<.001	1	>.99
D012: App use at work	0	1	>.99		1.2	1	.28
D013: App recommended	2.6	1	.11		3.0	1	.08
A303^d^: App known to participants	0.5	2	.77		2.4	2	.30

^a^A1: assessment 1.

^b^A2: assessment 2.

^c^Degrees of freedom may differ between both groups because, for either group, there may be categories with a row sum of zero.

^d^This question was shown on page 7 of assessment 3 (A3) and was not part of the demographics part of the survey on page 8; therefore, it was encoded differently.

**Table 13:** Assessment of whether there was sufficient information matching the nine quality principles versus changes in assessment: initial to intermediate assessment (ie, changes from A1^a^ to A2^b^ toward a more critical assessment).

Quality principle (as listed in questionnaire 2 [Q2]) vs A1→A2	Group A			Group B		
	χ²	df	*P* value	χ²	df	*P* value
Q201: Practicality	8.6	2	.01	1.1	2	.59
Q202: Risk adequacy	15.0	2	<.001	3.2	2	.21
Q203: Ethical soundness	9.0	2	.01	3.8	2	.15
Q204: Legal conformity	0.043	2	.98	5.9	2	.05
Q205: Content validity	4.3	2	.11	6.9	2	.03
Q206: Technical adequacy	1.1	2	.57	3.5	2	.17
Q207: Usability	3.8	2	.15	0.9	2	.65
Q208: Resource efficiency	0.1	2	.94	8.4	2	.02
Q209: Transparency	2.4	2	.30	9.7	2	.01

^a^A1: assessment 1.

^b^A2: assessment 2.

## Discussion

### Principal Findings

#### Confrontation With the Quality Principles Influences Participants’ App Usage Decisions

For groups A and B, there were statistically significant changes in the physicians’ assessments toward a more critical appraisal of the sufficiency of app descriptions for their individual app usage decisions after confronting the nine quality principles (see contingency Table 8 listing the participants’ decisions for A1 vs A2). Cumulative changes from *do not know* to *insufficient* and changes from *sufficient* to either *do not know* or *insufficient* occurred for 28.6% (63/220) of participants in group A (McNemar-Bowker test [df=3]=42.89, *P*<.001, Cohen g=0.39). Cumulative changes from *do not know* to *insufficient* and changes from *sufficient* to either *do not know* or *insufficient* occurred for 28.1% (62/221) of participants from group B (McNemar-Bowker test [df=3]=49.06, *P*<.001, Cohen g=0.42). The effect was very strong in both groups. There was a less pronounced, but statistically insignificant, effect regarding changes in opinion toward a more critical appraisal after applying the 25 questions (Q3) to the app descriptions (see contingency Table 11 showing the participants’ decisions for A2 vs A3). Opinions changed toward a more critical appraisal for 11.4% (25/220) of participants in group A (McNemar-Bowker test [df=3]=5.67, *P*=.13, Cohen g=0.19). Opinions changed toward a more critical appraisal for 9.0% (20/221) of participants in group B (McNemar-Bowker test [df=3]=7.66, *P*=.05, Cohen g=0.23). The similarly comprehensible change in assessment in both groups can be attributed to increased awareness about, and confrontation with, the quality principles and criteria, as well as their characteristics and requirements. Working with the nine generic criteria in Q1 and Q2 already led to changes in almost one-third of the cases. In contrast, the more detailed assessments performed in the subsequent step of the study (Q3), based on the 25 questions, only led to changes in assessment in just under 10% of cases (see Table 10). This suggests that even contemplating one's own prioritization of quality principles has a major effect on decision making. This also suggests that the more elaborate and detailed evaluation based on the 25 filter questions provided in a checklist format might have a supplementary effect. Assuredly, the latter tool is also well suited for the assessment process, but its completion requires considerably more time and effort. Also, it is less flexible than the generic quality principles. The question of which of the two instruments, or whether the combination of both, is more promising will need to be addressed in future studies, especially when it comes to questions of acceptance of the tools presented here and their economy of use.

#### Quality Principles Are Relevant

The statements among the two investigated groups differed regarding which quality principles were associated with a conservative appraisal (see Table 13). In group A, the quality principles *practicality* (*P*=.01), *risk adequacy* (*P*<.001), and *ethical soundness* (*P*=.01) correlated with a conservative appraisal. However, in group B, there was a statistically significant association with a conservative appraisal for *content validity* (*P*=.03), *resource efficiency* (*P*=.02), and *transparency* (*P*=.01). With the exception of one characteristic in group B, groups A and B did not differ significantly when considering demographics versus changes in assessment from A1 to A2 (see Table 12). As well, the evaluation of the basic relevance for the usage decision did not differ in any characteristic (see Figure 2 and Multimedia Appendix 1). This suggests that either the app descriptions or the random allocation of highly heterogeneous app descriptions led to this result.

Regarding sufficiency of information for the nine quality principles, we wanted to know which of these principles accounted for the largest proportion of the changes in decisions. Based on the regression analysis, contributions of the nine principles to changes in usage decisions differed between both groups (see Multimedia Appendix 4). However, the validity of the models was relatively weak, with a Nagelkerke’s R^2^ ranging from .051 to .085. Therefore, follow-up studies need to be carried out to further examine how much each variable may possibly contribute.

#### Insufficient Information Quality of the App Descriptions

It can be assumed that the lack of information available in the app descriptions was the most important factor influencing the results of the correlations and regression models. Descriptively, the poor information quality could be mapped to both the nine generic quality principles and the 25 filter questions. Again, groups A and B did not differ significantly in their distribution. Information on the nine quality principles could only be identified for the given principles in about 5%-20% of the cases (see Multimedia Appendix 2). The exception was for the quality principle *practicality*, for which there was information deemed sufficient for decision making, discernible from the app descriptions in 45.5% and 49.3% of the individual cases in group A and group B, respectively. The more detailed examination, performed by means of the 25 filter questions, confirmed this observation (see Multimedia Appendix 3). Five attributes were assigned to the quality principle *practicality* (see Table 4). While information on *specification of purpose* was found in about 86% of the cases (group A: 191/220, 86.8%; group B: 190/221, 86.0%), information on functionality in this context was only available in just 60% of the cases (see Multimedia Appendix 3; group A: 125/220, 56.8%; group B: 136/221, 61.5%). The situation for *methods that are applied* (group A: 30/220, 13.6%; group B: 25/221, 11.3%), the *proofs regarding the assigned purpose* (group A: 22/220, 10.0%; group B: 25/221, 11.3%), and *suitability* (group A: 25/220, 11.4%; group B: 20/221, 9.0%) was considerably worse. Here, information was found for less than 15% of the cases. Apart from a few exceptions, less than 10% of the other filter questions for both groups lead to information on the respective aspects being identified. Exceptions to this were isolated aspects such as *target group* (group A: 75/220, 34.1%; group B: 75/221, 33.9%), *quality of content* (group A: 32/220, 14.5%; group B: 34/221, 15.4%), *scope of information* (group A: 33/220, 15.0%; group B: 37/221; 16.7%), and *validity of information* (group A: 22/220, 10.0%; group B: 35/221, 15.8%). Thus, the presentation of information stands in stark contrast to the assessment of the importance of the quality principles for the decision on use.

#### Physicians Predominantly Perceive All Quality Principles as Important

Initially, the physicians considered the nine criteria to be largely *important* or *very important* for their usage decision (see Multimedia Appendix 1), with *content validity* (group A: 217/220, 98.6%; group B: 215/220, 97.3%), *risk adequacy* (group A: 206/220, 93.6%; group B: 196/221, 88.7%), and *legal conformity* (group A: 203/220, 92.3%; group B: 199/221, 90.0%) receiving the greatest approval. *Resource efficiency* (group A: 142/220, 64.5%; group B: 128/221, 57.9%) ranked last. This contrasts strongly with the information actually found in the app descriptions. It was only for *practicality* (group A: 188/220, 85.5%; group B: 191/221, 86.4%) that the necessary information could be identified in about half of the apps. For all other criteria, the corresponding information was available in less than one-fifth of the provided app descriptions.

### Comparison With the Previous Study

The results presented here confirm those of our previous study conducted with a comparable design, although with medical students [9]. A more detailed analysis will be presented in a subsequent article, although the core aspects will be outlined here. In our previous study, it was possible to show that an exploration of quality principles already influences individual usage decisions that are made based on app descriptions. Based on the results presented in this study, however, it could be shown that this influence can largely be attributed to nine generic principles and that working with these nine principles resulted in the greater proportion of changes for the usage decisions. Due to the design of the predecessor study, it was impossible to show this effect previously. The information concerning the identification of specific information from the app descriptions in the context of both studies was comparably sobering. In 83.17% of the cases (3301/3969 ratings for all nine quality principles in Q2; cumulative answers for *no* and *do not know*), the physicians included in this study were unable to find sufficient information for the nine principles, while among the students, this was the case in 80% [9] of the submitted ratings. Descriptively, physicians and students seem to agree with each other in their requirements as to which quality principles are of relevance for the individual decision on use. Each of the nine quality principles was predominantly regarded as *very important or important*, with *resource efficiency* still receiving the lowest percentage of approval among both populations (group A: 64.5%; group B: 57.9%; medical students from previous study: 63.4%). One difference was found for the principle of *technical adequacy*, which physicians in both groups predominantly regarded as *very important or important*, while students viewed it more cautiously (group A: 83.6%; group B: 84.6%; medical students from previous study: 41.4%). It is possible that these rather technical aspects were too abstract for the target groups in the quality context.

As far as identifying information that matches the generic principles is concerned, there is agreement between physicians and students that if information is available, it is most often found for the quality principle of *practicality*. The physicians, however, were stricter in their assessment than the students from the previous study (group A: 44.5%; group B: 49.3%; medical students from previous study: 71.7%). In a follow-up study, the comparability of student and physician attitudes will be examined in more detail in order to further investigate the validity of the differences and their causes.

### Limitations

Due to the study's design, the selection of apps was limited to cardiological and pulmonological apps only. The identical set of apps that was employed in the previous study was used and no changes were made to the selection. Therefore, the limitations concerning the app selection correspond to those of the previous study [9]. The actuality of the apps does not matter, since archived app descriptions were used.

The study population included only members of the DGIM who were willing to participate and is, thus, certainly not representative of the entire medical profession and not even representative of the more than 21,000 members of the association. However, the sole recruitment method via this society was chosen since control mechanisms, such as targeted but anonymous addressing of physicians, involvement of stakeholders, suitable communication channels, and recruitment instruments of this society, could be used to obtain the required number of cases. However, the authors assume that the examination of quality aspects relating to software is independent of the discipline. This is a conclusion based on our observations and experiences as well as the participation of one of the authors in numerous initiatives of different medical societies, where identical problems are likewise debated.

Despite the large number of invitations that were sent out, only a relatively small number of individuals (N=724) chose to start the survey. Of those, only 441 completed all pages of the survey, with varying numbers of dropouts at different stages (see Table 5); an additional 15 had not given their consent and stated so on the introductory page. For those who simply stopped answering the survey, there was no way to determine the reasons for not continuing; some of the free-text comments given by those who had persevered may still provide insight into possible reasons for not proceeding. For some participants, it may not have been clear that the different approaches presented for evaluation at different stages might influence their opinion; in their final comments after having already finished the survey, they therefore voiced their discontent about seemingly having to answer similar questions (eg, the usage decisions in A1-A3) multiple times. Others were highly frustrated by some of the presented app descriptions, specifically regarding content and language (ie, spelling and grammar), also stating that they were unable to see the relevance of the randomly assigned app description for their line of work. There was also a statement to the effect that the presented criteria and questions were too extensive and time-consuming. For future work, the latter point may possibly be addressed based on our results: while working with the nine criteria had a definite influence on the usage decision, the effect of the 25 detailed key questions was negligible; for those who want to quickly come to a decision, applying the nine base criteria may therefore suffice. We believe these criteria to be a valuable tool in order to not forget important aspects when weighing the pros and cons of using an app, although there were also some participants who stated that simply installing and testing an app would suffice.

All nine criteria were rated mostly *important* or *very important* by the physicians. Therefore, it is difficult to significantly identify a hierarchy of relevance. This aspect will be addressed in a follow-up study that will apply designs from requirements analysis, as prioritization of aspects with supposedly identical relevance is a known issue of requirements engineering.

Our sample size considerations have only partially been met. Detecting a difference between A1 and A2 was covered with our initial assumptions; however, the comparison of A2 and A3 would have required a larger sample size to detect a difference, a fact that we were not aware of at the planning stage.

The questionnaire design was adopted from the previous study and was revised in a detailed pretest (see subsection *Pretest of the Questionnaires* under Methods). In particular, the comprehensibility of the questions and tasks, as well as usability aspects related to the electronic system, were dealt with in order to create the greatest possible comfort for the participants. However, the 25 detailed questions could not be reworded with regard to their complexity, as they corresponded to the wording of a technical document [20]. The objective here was to avoid any falsification. Nevertheless, while in the pretest, the questions and tasks were all interpreted and paraphrased correctly, it cannot be ruled out that the questions might have been too complex for fast readers.

With the given setup, we could not assess test-retest reliability. It would have been an interesting aspect to determine whether participants would have given the same answer under similar conditions.

### Outlook

In line with the aim of this study, specifically the effect of sensitization to quality aspects of health software—health apps in particular—the following work is being carried out: (1) an evaluation of the existing dataset with respect to questions of acceptance, (2) a detailed comparison of the preceding study with medical students with the question of whether there are differences in attitude and evaluation between medical students and physicians, (3) a study to determine which combination of quality principles, particularly filter criteria, has the greatest acceptance among the target group, and (4) a study that examines the app selections that were used and tries to determine which information should be searched for beyond app descriptions in order to be able to assess the relevance of the descriptions in relation to other information sources.

### Conclusions

The study confirms that sensitizing physicians to the topic of quality principles via questions about attitudes toward established quality principles and their applications results in a more critical evaluation of the sufficiency of app descriptions. Even working with only nine generic criteria was sufficient to bring about the majority of decision changes, while the additional, checklist-like processing of 25 detailed requirements contributed only slightly to the overall share of decision changes. All physicians shared their views on the relevance of the nine quality principles, and the predominant opinion was that these principles are *important* or *very important*. *Content validity*, *risk adequacy*, and *legal conformity* received the greatest approval. However, in the app descriptions themselves, it was rarely possible to identify any specific information matching these principles. At best, for *practicality*, such data were detected in about 50% of the descriptions. In up to 85% of the other eight criteria, no meaningful information could be identified.

## Appendix

Multimedia Appendix 1 Comparison of how the participants assessed the relevance of the nine quality principles (questionnaire 1 [Q1]) for their own usage decision (group A: N=220; group B: N=221).

Multimedia Appendix 2 Assessment as to whether information for the nine quality principles could be found within the available app descriptions: assessments for group A (N=220) and for group B (N=221).

Multimedia Appendix 3 Assessment of whether there was sufficient information to answer 25 detailed questions on the basis of the available app descriptions: assessments for group A (N=220) and group B (N=221).

Multimedia Appendix 4 Table D-1 (group A): Results of the regression analysis for group A for changes from assessment 1 (A1) to a more critical assessment in assessment 2 (A2) versus sufficiency of the information provided for assessing the nine quality principles; Table D-2 (group B): Results of the regression analysis for group B for changes from A1 to a more critical assessment in A2 versus sufficiency of the information provided for assessing the nine quality principles. Reference category for both tables was “no.” NR²: Nagelkerke’s R squared.

## Abbreviations

A1: assessment 1
A2: assessment 2
A3: assessment 3
D0: participant demographic
DGIM: Deutsche Gesellschaft für Innere Medizin
DPRS: discordant proportion ratio sum
OR: odds ratio
PASS: Power Analysis and Sample Size
Q1: questionnaire 1
Q2: questionnaire 2
Q3: questionnaire 3

## References

[1] Albrecht UV, Höhn M, von Jan U, Albrecht UV (2016). Gesundheits-Apps und markt. Chancen und Risiken von Gesundheits-Apps (CHARISMHA) [Chances and Risks of Mobile Health Apps (CHARISMHA)].

[2] Kebede M, Steenbock B, Helmer SM, Sill J, Möllers T, Pischke CR (2018). Identifying evidence-informed physical activity apps: Content analysis. JMIR Mhealth Uhealth.

[3] Yu L, Luo X, Qian C, Wang S, Leung HKN (2018). Enhancing the description-to-behavior fidelity in Android apps with privacy policy. IEEE Trans Softw Eng.

[4] Lee G, Raghu TS, Park S (2015). Do app descriptions matter? Evidence from mobile app product descriptions. SSRN.

[5] Han W, Wang W, Zhang X, Peng W, Fang Z (2015). APP betting based on the consistency of description and APK. Proceedings of the International Conference on Trusted Systems 2014 (INTRUST 2014).

[6] Buijink AW, Visser BJ, Marshall L (2013). Medical apps for smartphones: Lack of evidence undermines quality and safety. Evid Based Med.

[7] Hillebrand U, von Jan U, Albrecht UV (2016). Concepts for quality assurance of health-related apps. Stud Health Technol Inform.

[8] Albrecht UV, Hillebrand U, von Jan U (2018). Relevance of trust marks and CE labels in German-language store descriptions of health apps: Analysis. JMIR Mhealth Uhealth.

[9] Albrecht U, Malinka C, Long S, Raupach T, Hasenfuß G, von Jan U (2019). Quality principles of app description texts and their significance in deciding to use health apps as assessed by medical students: Survey study. JMIR Mhealth Uhealth.

[10] Scott IA, Scuffham P, Gupta D, Harch TM, Borchi J, Richards B (2018). Going digital: A narrative overview of the effects, quality and utility of mobile apps in chronic disease self-management. Aust Health Rev.

[11] Grundy QH, Wang Z, Bero LA (2016). Challenges in assessing mobile health app quality: A systematic review of prevalent and innovative methods. Am J Prev Med.

[12] (2017). Ministry of Health – Manatū Hauora.

[13] (2015). Guiding Principles for Physicians Recommending Mobile Health Applications to Patients.

[14] (2019). Digital Assessment Questions V2.2.

[15] (2018). Fraunhofer-Institut für Offene Kommunikationssysteme FOKUS.

[16] Albrecht UV (2019). Transparenz schaffen und Orientierung bieten: Methoden und Werkzeuge als Entscheidungshilfe für die Nutzung von Gesundheits-Apps. Erstellung einer ersten Auslegeordnung zur Entwicklung eines Hilfsmittels für schweizerische Anwender.

[17] Hennemann S, Beutel ME, Zwerenz R (2017). Ready for eHealth? Health professionals' acceptance and adoption of eHealth interventions in inpatient routine care. J Health Commun.

[18] Martínez-Pérez B, de la Torre-Díez I, Candelas-Plasencia S, López-Coronado M (2013). Development and evaluation of tools for measuring the quality of experience (QoE) in mHealth applications. J Med Syst.

[19] Tarricone R, Cucciniello M, Armeni P, Petracca F, Desouza KC, Hall LK, Keefe D (2019). Mobile health divide between clinicians and patients in cancer care: Results from a cross-sectional international survey. JMIR Mhealth Uhealth.

[20] Albrecht UV (2019). Einheitlicher Kriterienkatalog zur Selbstdeklaration der Qualität von Gesundheits-Apps.

[21] SoSci Survey.

[22] McNemar Q (1947). Note on the sampling error of the difference between correlated proportions or percentages. Psychometrika.

[23] Bowker AH (1948). A test for symmetry in contingency tables. J Am Stat Assoc.

[24] (2018). PASS Sample Size Software.

[25] Chow SC, Shao J, Wang H (2008). Sample Size Calculations in Clinical Research. 2nd edition.

[26] Wickham H, François R, Henry L, Müller K (2019). The Comprehensive R Archive Network.

[27] Wickham H (2016). ggplot2: Elegant Graphics for Data Analysis.

[28] Neuwirth E (2014). The Comprehensive R Archive Network.

[29] Heinzen E, Sinnwell J, Atkinson E, Gunderson T, Dougherty G (2019). The Comprehensive R Archive Network.

[30] DeWitt P (2019). The Comprehensive R Archive Network.

[31] Harrell FE Jr, Dupont C (2019). The Comprehensive R Archive Network.

[32] Signorell A (2019). The Comprehensive R Archive Network.

[33] Mangiafico S (2019). The Comprehensive R Archive Network.

[34] (2019). The R Project for Statistical Computing.

