# Supplementary material for: Quality Awareness and Its Influence on the Evaluation of App Meta-Information by Physicians: Validation Study
Source: JMIR Mhealth Uhealth. 2019 Nov 18;7(11):e16442. doi: 10.2196/16442 (PMC6887815; doi:10.2196/16442)
Supplement: Multimedia Appendix 1 [file mhealth_v7i11e16442_app1.pdf]

Multimedia Appendix 1

Table A: Comparison of how the participants assessed the relevance of the nine quality principles (question 1 [Q1]) for their own usage decision (group A: N=220; group B: N=221).

		**Group A (N=220), n (%)**	**Group B (N=221), n (%)**	χ^2	**df**	***P***
**Q101: Practicality** The software can be used for the intended purpose and must be as versatile as possible in order to open up the largest possible application areas and contexts.				0.4	3	0.94
	Very important or important	188 (85.5)	191 (86.4)			
	Partly important	17 (7.7)	14 (6.3)			
	Less important or unimportant	11 (5.0)	11 (5.0)			
	Do not know	4 (1.8)	5 (2.3)			
**Q102: Risk adequacy** The software provides the means to be used in a risk-appropriate manner without exposing the user or his or her environment to a disproportionate health, social, or economic risk.				6.4	3	0.09
	Very important or important	206 (93.6)	196 (88.7)			
	Partly important	13 (5.9)	17 (7.7)			
	Less important or unimportant	1 (0.5)	5 (2.3)			
	Do not know	0 (0.0)	3 (1.4)			
**Q103: Ethical soundness** Development, provision, operation, and use of the software are ethically innocuous in order to prevent discrimination and stigmatization and to facilitate fair access.				5.1	3	0.16
	Very important or important	190 (86.4)	179 (81.0)			
	Partly important	18 (8.2)	27 (12.2)			
	Less important or unimportant	12 (5.5)	12 (5.4)			
	Do not know	0 (0.0)	3 (1.4)			
**Q104: Legal conformity** Legal conformity (eg, medical device law, professional law, data protection law, and law on the advertising of therapeutic products) of the development, provision, operation, and use of the software is guaranteed for the protection of all parties involved (eg, providers, store operators, and users).				2.4	3	0.49
	Very important or important	203 (92.3)	199 (90.0)			
	Partly important	10 (4.5)	13 (5.9)			
	Less important or unimportant	7 (3.2)	7 (3.2)			
	Do not know	0 (0.0)	2 (0.9)			
**Q105: Content validity** The health-related content of the software that is presented and used is valid and trustworthy (ie, scientifically sound, up-to-date, and without conflict of interest).				1.7	3	0.64
	Very important or important	217 (98.6)	215 (97.3)			
	Partly important	2 (0.9)	4 (1.8)			
	Less important or unimportant	1 (0.5)	1 (0.5)			
	Do not know	0 (0.0)	1 (0.5)			
**Q106: Technical adequacy** Development, operation, maintenance, and use of the software correspond to the state of the art in order to enable sustainability in terms of maintainability as well as platform-independent and cross-platform use (eg, in terms of portability of the app or interoperability or compatibility with other products).				0.7	3	0.87
	Very important or important	184 (83.6)	187 (84.6)			
	Partly important	29 (13.2)	25 (11.3)			
	Less important or unimportant	6 (2.7)	7 (3.2)			
	Do not know	1 (0.5)	2 (0.9)			

**Q107: Usability**<br>The software allows the target group to make appropriate use of it (eg, through product ergonomics, accessibility, and aesthetics), which contributes to user satisfaction.				1.1	3	0.77
	Very important or important	191 (86.8)	190 (86.0)			
	Partly important	18 (8.2)	23 (10.4)			
	Less important or unimportant	10 (4.5)	7 (3.2)			
	Do not know	1 (0.5)	1 (0.5)			
**Q108: Resource efficiency**<br>During development of the software, elements for resource-efficient operation (eg, energy consumption) and use (eg, computing time) are taken into account.				2.3	3	0.52
	Very important or important	142 (64.5)	128 (57.9)			
	Partly important	53 (24.1)	61 (27.6)			
	Less important or unimportant	24 (10.9)	30 (13.6)			
	Do not know	1 (0.5)	2 (0.9)			
**Q109: Transparency**<br>Complete transparency regarding the quality principles serves as a basis for evaluations of the software as well as for individual and collective usage decisions.				4.2	3	0.24
	Very important or important	191 (86.8)	176 (79.6)			
	Partly important	21 (9.5)	32 (14.5)			
	Less important or unimportant	5 (2.3)	9 (4.1)			
	Do not know	3 (1.4)	4 (1.8)			
