# Supplementary material for: Quality Awareness and Its Influence on the Evaluation of App Meta-Information by Physicians: Validation Study
Source: JMIR Mhealth Uhealth. 2019 Nov 18;7(11):e16442. doi: 10.2196/16442 (PMC6887815; doi:10.2196/16442)
Supplement: Multimedia Appendix 2 [file mhealth_v7i11e16442_app2.pdf]

Multimedia Appendix 2

Table B. Assessment as to whether information for the 9 quality principles could be found within the available app descriptions: assessments for group A (N=220) and for group B (N=221).

	**Group A (N=220), n (%)**	**Group B (N=221), n (%)**	χ^2	**df**	***P***
Q201: Practicality: Can you use the app description to make an assessment as to whether the app is useful?			1.1	2	.59
Yes	98 (44.5)	109 (49.3)			
No	100 (45.5)	93 (42.1)			
Do not know	22 (10.0)	19 (8.6)			
Q202: Risk-adequacy: Can you use the app description to make an assessment as to whether the app is risk-adequate?			2	2	.36
Yes	29 (13.2)	40 (18.1)			
No	162 (73.6)	153 (69.2)			
Do not know	29 (13.2)	28 (12.7)			
Q203: Ethical soundness: Can you use the app description to make an assessment as to whether the app is ethically safe?			0.5	2	.07
Yes	31 (14.1)	30 (13.6)			
No	160 (72.7)	176 (79.6)			
Do not know	29 (13.2)	15 (6.8)			
Q204: Legal conformity: Can you use the app description to make an assessment as to whether the app is legally compliant?			0.5	2	.76
Yes	21 (9.5)	17 (7.7)			
No	173 (78.6)	179 (81.0)			
Do not know	26 (11.8)	25 (11.3)			
Q205: Content validity: Can you use the app description to make an assessment as to whether the content of the app is valid?			1.3	2	.51
Yes	38 (17.3)	37 (16.7)			
No	163 (74.1)	171 (77.4)			
Do not know	19 (8.6)	13 (5.9)			
Q206: Technical adequacy: Can you use the app description to make an assessment as to whether the app is technically appropriate?			3.8	2	.15
Yes	25 (11.4)	38 (17.2)			
No	167 (75.9)	162 (73.3)			
Do not know	28 (12.7)	21 (9.5)			
Q207: Usability: Can you use the app description to make an assessment as to whether the app is usable?			0.9	2	.64
Yes	40 (18.2)	48 (21.7)			
No	156 (70.9)	149 (67.4)			
Do not know	24 (10.9)	24 (10.9)			
Q208: Resource efficiency: Can you use the app description to make an assessment as to whether the app is resource-efficient?			0.8	2	.66
Yes	16 (7.3)	12 (5.4)			
No	188 (85.5)	190 (86.0)			
Do not know	16 (7.3)	19 (8.6)			
Q209: Transparency: Can you use the app description to make an assessment as to whether the app description is transparent on the above points?			0.2	2	.90
Yes	20 (9.1)	19 (8.6)			
No	178 (80.9)	177 (80.1)			
Do not know	22 (10.0)	25 (11.3)			
