# Supplementary material for: Quality Awareness and Its Influence on the Evaluation of App Meta-Information by Physicians: Validation Study
Source: JMIR Mhealth Uhealth. 2019 Nov 18;7(11):e16442. doi: 10.2196/16442 (PMC6887815; doi:10.2196/16442)
Supplement: Multimedia Appendix 3 [file mhealth_v7i11e16442_app3.pdf]

Multimedia Appendix 3

Table C. Assessment of whether there was sufficient information to answer 25 detailed questions on the basis of the available app descriptions: assessments for group A (N=220) and group B (N=221).

		Group A (N=220), n (%)	Group B (N=221), n (%)	χ^2	df	*P*
Q301: Has the purpose of the app been specified?				2.8	2	.25
	Yes	191 (86.8)	190 (86.0)			
	No	19 (8.6)	26 (11.8)			
	Do not know	10 (4.5)	5 (2.3)			
Q302: Is there a description of which functions the app includes to fulfill its purpose?				3.6	2	.17
	Yes	125 (56.8)	136 (61.5)			
	No	86 (39.1)	70 (31.7)			
	Do not know	9 (4.1)	15 (6.8)			
Q303: Is there a description of the methods employed by the app to fulfill its purpose given (e.g. procedures, processes, algorithms with which the offered functions are implemented) and are there statements regarding their suitability for this purpose?				1.9	2	.38
	Yes	30 (13.6)	25 (11.3)			
	No	177 (80.5)	176 (79.6)			
	Do not know	13 (5.9)	20 (9.0)			
Q304: Is appropriate evidence cited to support the statements on fulfillment of the purpose of the app (e.g. references to studies, guidelines, testing, and quality labels)?				1.0	2	.62
	Yes	22 (10.0)	25 (11.3)			
	No	188 (85.5)	182 (82.4)			
	Do not know	10 (4.5)	14 (6.3)			
Q305: Are the suitability and unsuitability for certain application scenarios or user groups specified (e.g. in terms of inclusion and exclusion criteria)?				0.5	2	.78
	Yes	25 (11.4)	30 (13.6)			
	No	179 (81.4)	175 (79.2)			
	Do not know	16 (7.3)	16 (7.2)			
Q306: Have potential or actual risks the app poses to users or their environment (with respect to health, economic, or social aspects) been stated?				1.5	3	.68
	Yes	14 (6.4)	11 (5.0)			
	Not answered	1 (0.5)	0 (0.0)			
	No	193 (87.7)	199 (90.0)			
	Do not know	12 (5.5)	11 (5.0)			
Q307: Have precautions been taken to avoid health, economic and/or social risks when using the described app?				2	2	.37
	Yes	21 (9.5)	14 (6.3)			
	No	186 (84.5)	190 (86.0)			
	Do not know	13 (5.9)	17 (7.7)			
Q308: Is there a description about the extent to which the app follows ethical principles such as patient autonomy, equity of access and/or professional ethics and research ethics?				1.5	2	.47
	Yes	9 (4.1)	10 (4.5)			
	No	195 (88.6)	201 (91.0)			
	Do not know	16 (7.3)	10 (4.5)			
Q309: Are conflicts of interest (e.g. authors with affiliation to specific companies) discussed in the app description text?				0.6	2	.74
	Yes	12 (5.5)	10 (4.5)			
	No	199 (90.5)	199 (90.0)			
	Do not know	9 (4.1)	12 (5.4)			
Q310: Is there a mention of whether the app is being provided in a research context and if so, is there a statement about whether it follows good scientific practice?				0.8	2	.68
	Yes	18 (8.2)	22 (10.0)			
	No	187 (85.0)	181 (81.9)			
	Do not know	15 (6.8)	18 (8.1)			

		Group A (N=220), n (%)	Group B (N=221), n (%)	χ^2	df	*P*
Q311: Is there a statement about whether the relevant general legal requirements, such as data protection law, telemedia law, commercial law, have been taken into account by the manufacturer/provider of the app?				0.6	2	.73
	Yes	12 (5.5)	16 (7.2)			
	No	191 (86.8)	187 (84.6)			
	Do not know	17 (7.7)	18 (8.1)			
Q312: Is there a statement about which requirements/regulations have been taken into account with regard to using the app in a health context, such as medical device law or medical professional law?				0.5	2	.76
	Yes	16 (7.3)	13 (5.9)			
	No	189 (85.9)	195 (88.2)			
	Do not know	15 (6.8)	13 (5.9)			
Q313: Is there a statement about how the quality of the content has been ensured (e.g. involvement of experts in the field) or which (validated) sources have been used (e.g. consideration of current scientific findings, guidelines)?				1.1	3	.79
	Yes	32 (14.5)	34 (15.4)			
	No	171 (77.7)	171 (77.4)			
	Do not know	16 (7.3)	16 (7.2)			
	Not answered	1 (0.5)	0 (0.0)			
Q314: Is there a description of how the app is regularly adapted to new content requirements?				2.2	3	.53
	Yes	21 (9.5)	19 (8.6)			
	No	182 (82.7)	188 (85.1)			
	Do not know	15 (6.8)	14 (6.3)			
	Not answered	2 (0.9)	0 (0.0)			
Q315: Is it described to what extent the app corresponds to the current state of the art?				1.4	3	.71
	Yes	17 (7.7)	14 (6.3)			
	No	185 (84.1)	188 (85.1)			
	Do not know	17 (7.7)	19 (8.6)			
	Not answered	1 (0.5)	0 (0.0)			
Q316: It there information about how the app is regularly adapted to technical requirements?				1.1	3	.77
	Yes	14 (6.4)	14 (6.3)			
	No	192 (87.3)	192 (86.9)			
	Do not know	13 (5.9)	15 (6.8)			
	Not answered	1 (0.5)	0 (0.0)			
Q317: Is there information about to what extent it is possible to switch to another operating system or device without data loss?				1.4	3	.71
	Yes	6 (2.7)	7 (3.2)			
	No	201 (91.4)	195 (88.2)			
	Do not know	12 (5.5)	18 (8.1)			
	Not answered	1 (0.5)	1 (0.5)			
Q318: Is there a mention of whether the app is scalable (adaptable to increasing requirements) or can be integrated into other products?				0.8	3	.85
	Yes	12 (5.5)	14 (6.3)			
	No	195 (88.6)	190 (86.0)			
	Do not know	12 (5.5)	16 (7.2)			
	Not answered	1 (0.5)	1 (0.5)			
Q319: Has information been provided about proofs for the app's usability (e.g. usability tests)?				1.9	3	.59
	Yes	13 (5.9)	7 (3.2)			
	No	190 (86.4)	196 (88.7)			
	Do not know	16 (7.3)	17 (7.7)			
	Not answered	1 (0.5)	1 (0.5)			

		Group A (N=220), n (%)	Group B (N=221), n (%)	χ^2	df	*P*
Q320: Is it described to what extent the function of the app has been specifically adapted to the target group, is barrier-free or can be used with individual adaptations?				4.4	3	.22
	Yes	18 (8.2)	20 (9.0)			
	No	178 (80.9)	189 (85.5)			
	Do not know	22 (10.0)	11 (5.0)			
	Not answered	2 (0.9)	1 (0.5)			
Q321: Is there information about the extent to which user feedback was considered for the app (e.g. during the development process)?				2.1	3	.55
	Yes	16 (7.3)	11 (5.0)			
	No	185 (84.1)	196 (88.7)			
	Do not know	17 (7.7)	13 (5.9)			
	Not answered	2 (0.9)	1 (0.5)			
Q322: Is there a statement about how the app ensures efficient use of the available technical resources (e.g. required memory, computing power, internal or external sensors, power consumption, etc.)?				2.8	3	.43
	Yes	7 (3.2)	7 (3.2)			
	No	204 (92.7)	197 (89.1)			
	Do not know	8 (3.6)	16 (7.2)			
	Not answered	1 (0.5)	1 (0.5)			
Q323: Is the information about the app sufficient, i.e. adequately specified in scope and depth of information?				0.9	3	.82
	Yes	33 (15.0)	37 (16.7)			
	No	168 (76.4)	161 (72.9)			
	Do not know	18 (8.2)	21 (9.5)			
	Not answered	1 (0.5)	2 (0.9)			
Q324: Is valid information (i.e. complete and reliable) given about the app?				4.5	3	.21
	Yes	22 (10.0)	35 (15.8)			
	No	168 (76.4)	150 (67.9)			
	Do not know	29 (13.2)	35 (15.8)			
	Not answered	1 (0.5)	1 (0.5)			
Q325: Is the information about the app described in a manner that is adequate for the target group?				0.7	3	.87
	Yes	75 (34.1)	75 (33.9)			
	No	118 (53.6)	113 (51.1)			
	Do not know	26 (11.8)	32 (14.5)			
	Not answered	1 (0.5)	1 (0.5)			
