# Supplementary material for: Quality Awareness and Its Influence on the Evaluation of App Meta-Information by Physicians: Validation Study
Source: JMIR Mhealth Uhealth. 2019 Nov 18;7(11):e16442. doi: 10.2196/16442 (PMC6887815; doi:10.2196/16442)
Supplement: Multimedia Appendix 4 [file mhealth_v7i11e16442_app4.pdf]

Multimedia Appendix 4

Regression Analysis

Group A (N=220)

Table D-1: Results of the regression analysis for group A for changes from A1 to a more critical assessment in A2 versus sufficiency of the information provided for assessing the 9 quality principles. Reference category was "no". NR^2 = Nagelkerke's R squared.

**Criterion**	**Direction**	***P***	**OR**	**CI95 low**	**CI95 high**	**NR^2**
**Q201: Practicality**						
	No					
	Do not know	0.189	1.989	0.712	.555	.056
	Yes	.004	2.586	1.356	4.930	.056
	Constant	<.001	.235			
**Q202: Risk adequacy**						
	No					
	Do not know	<.001	4.321	1.902	9.818	.085
	Yes	.643	.796	0.303	2.092	.085
**Q203: Ethical soundness**						
	No					
	Do not know	.004	3.324	1.475	7.494	.053
	Yes	.585	1.269	.540	2.986	.053
	Constant	<.001	.322			
Q204: Legal conformity						
	No					
	Do not know	.835	.906	.359	2.290	<.001
	Yes	.975	.984	.361	2.681	<.001
	Constant	.000	.407			
**Q205: Content validity**						
	No					
	Do not know	.092	2.289	.874	5.996	.027
	Yes	.371	.678	.290	1.589	.027
	Constant	<.001	.393			
**Q206: Technical adequacy**						
	No					
	Do not know	.764	1.141	.483	2.696	
	Yes	.337	.602	.214	1.695	.008
	Constant	<.001	.415			.008
**Q207: Usability**						
	No					
	Do not know	.093	2.143	.881	5.212	.024
	Yes	.206	1.615	.768	3.399	.024
	Constant	<.001	.333			
**Q208: Resource efficiency**						
	No					
	Do not know	.795	1.158	.384	3.492	.001
	Yes	.795	1.158	.384	3.492	.001
	Constant	<.001	.393			
**Q209: Transparency**						
	No					
	Do not know	.218	1.772	.713	4.406	.015
	Yes	.444	.640	.204	2.008	0.15
	Constant	<.001	.391			

Group B (N=221)

Table D-2: Results of the regression analysis for group B for changes from A1 to a more critical assessment in A2 versus sufficiency of the information provided for assessing the 9 quality principles. Reference category was “no”. NR^2 = Nagelkerke’s R squared.

Criterion	Direction	P	OR	CI95 low	CI95 high	NR^2
**Q201: Practicality**						
	No					
	Do not know	.884	1.087	.353	3.347	.007
	Yes	.310	1.380	.741	2.568	.007
	Constant	<.001				
**Q202: Risk adequacy**						
	No					
	Do not know	.506	1.333	.571	3.112	.022
	Yes	.136	.509	.210	1.236	.022
	Constant					
**Q203: Ethical soundness**						
	No					
	Do not know	.746	.822	.250	2.696	.028
	Yes	.060	.348	.116	1.045	.028
	Constant	<.001	.3222			
**Q204: Legal conformity**						
	No					
	Do not know	.254	.549	.196	1.538	.046
	Yes	.057	.137	.018	1.061	.046
	Constant	<.001	.455			
**Q205: Content validity**						
	No					
	Do not know	.578	1.392	.435	4.454	.051
	Yes	.018	.270	.091	.801	.051
	Constant	<.001	.449			
**Q206: Technical adequacy**						
	No					
	Do not know	.249	.512	1.64	1.599	.024
	Yes	.116	.491	.203	1.190	.024
	Constant	<.001	.459			
**Q207: Usability**						
	No					
	Do not know	.454	.670	.235	1.912	.006
	Yes	.684	1.158	.571	2.348	.006
	Constant	<.001	.393			
**Q208: Resource efficiency**						
	No					
	Do not know	.042	.120	.016	.923	.067
	Yes	.124	.197	.025	1.561	.067
	Constant	<.001	.462			
**Q209: Transparency[a]**						
	No					
	Do not know	.264	.562	.205	1.544	.075
	Yes	.048	.054	.003	.974	
	Constant	<.001	.475			

[a] Firth's correction was applied due to a zero cell.
